# Competing risk and heterogeneity of treatment effect in clinical trials

**DOI:** 10.1186/1745-6215-9-30

**Published:** 2008-05-22

**Authors:** David M Kent, Alawi Alsheikh-Ali, Rodney A Hayward

**Affiliations:** 1 Institute for Clinical Research and Health Policy Studies, Department of Medicine, Tufts Medical Center, 750 Washington St, #63, Boston, MA, 02111, USA; 2 Cardiac Arrhythmia Center, Division of Cardiology, Department of Medicine, Tufts Medical Center, Tufts University School of Medicine, 750 Washington Street, NEMC #108, Boston, Massachusetts 02111, USA; 3 Veterans Affairs Ann Arbor Health Services Research and Development Service Center of Excellence; 4 Department of Internal Medicine, Universty of Michigan, 3110 Taubman Health Care Center, Ann Arbor, MI 48109-0368, USA

## Abstract

It has been demonstrated that patients enrolled in clinical trials frequently have a large degree of variation in their baseline risk for the outcome of interest. Thus, some have suggested that clinical trial results should routinely be stratified by outcome risk using risk models, since the summary results may otherwise be misleading. However, variation in competing risk is another dimension of risk heterogeneity that may also underlie treatment effect heterogeneity. Understanding the effects of competing risk heterogeneity may be especially important for pragmatic comparative effectiveness trials, which seek to include traditionally excluded patients, such as the elderly or complex patients with multiple comorbidities. Indeed, the observed effect of an intervention is dependent on the ratio of outcome risk to competing risk, and these risks – which may or may not be correlated – may vary considerably in patients enrolled in a trial. Further, the effects of competing risk on treatment effect heterogeneity can be amplified by even a small degree of treatment related harm. Stratification of trial results along both the competing and the outcome risk dimensions may be necessary if pragmatic comparative effectiveness trials are to provide the clinically useful information their advocates intend.

## Introduction

Recent commentaries have highlighted several fundamental limitations of clinical trials in providing an evidence-base for medical practice. It has been pointed out that many patients seen in routine clinical practice, particularly older and complex patients with multiple comorbid conditions, are excluded from clinical trials [1,2]. To address this, there has been a call for pragmatic comparative effectiveness trials with broader inclusion criteria, with the goal of enrolling a diverse patient population more representative of patients seen in routine clinical practice [3]. However, other commentaries have highlighted another limitation in clinical trials: substantial treatment-effect heterogeneity within trials often makes the overall summary result difficult to interpret and apply [4-7]. Enrolling a greater diversity of patients will increase this within trial heterogeneity. Thus, while some argue for broader inclusion criteria to make results more "generalizable", increasing patient heterogeneity yields overall results that are more likely to be uninformative or even misleading.

While a consensus has yet to fully emerge on how best to deal with treatment-effect heterogeneity, the limitations of conventional subgroup analyses are well-appreciated. Since patients have multiple attributes that might affect the risks and benefits of an intervention – they are male or female, young or old, with or without diabetes, have a high or low blood pressure, blood count, cholesterol, urinary protein excretion, ejection fraction, etc. – it is statistically impractical to consider each potentially important attribute in a one at a time manner [6,7]. It has therefore been suggested that patient characteristic be combined by risk models that describe fundamental dimensions of risk likely to underpin treatment-effect heterogeneity [6,9-11].

Prior work has demonstrated that variation in outcome-risk (i.e. a patient's baseline risk of having the outcome of interest) is a fundamental determinant of the opportunity for treatment benefit, and of the risk-benefit trade-offs when there is any treatment-related harm [6,9-11]. Because variation in outcome-risk among patients enrolled in clinical trials is ubiquitous, frequently large and typically skewed, a relatively small subgroup of high-risk patients often account for most trial outcomes and have a disproportionate influence on overall trial results [12]. Indeed, the summary result of a clinical trial might not even accurately reflect the tested intervention's treatment-effect in a typical patient within the trial [6,12]. Because of this, and because outcome-risk variation can often be well-described with a simple multivariate risk model, routine stratification of trial results by outcome-risk has been recommended [6,9-11,13]. In addition to outcome-risk, it is also recognized that, for treatments with serious and non-rare adverse effects (e.g. surgery or fibrinolytic therapy), individual patient variation in vulnerability to treatment-related harm can give rise to important treatment-effect heterogeneity; thus, it may in some circumstances be appropriate to stratify patients based on their risk of treatment-related harm [6,9,14,15].

However, another dimension of risk heterogeneity from which clinically significant differences in treatment-effect may emerge is relatively neglected and may be of particular importance for comparative effectiveness trials: variation in competing risk. Competing risk is the risk of an event that interferes with the probability of experiencing the disease-specific outcome of interest [16]. It is not merely a statistical issue affecting Kaplan-Meier [16] or sample size [17] estimates, but a clinical issue especially important when considering treatments in older or complex patients with multiple comorbidities for whom competing events may limit the likelihood of treatment benefit. This paper considers how – even for treatments with uniform treatment efficacy-understanding the complex interplay between baseline risk, treatment-related harm and competing risk is important in making good individual-patient recommendations and decisions, and how analyzing the effects of competing and outcome risks in clinical trials – normally obscured by overall trial results – may better inform clinical decision-making.

## Discussion

### When Competing Risk is Uncorrelated with Outcome-Risk

To illustrate the interplay between outcome-risk and competing risk, consider the use of adjuvant chemotherapy for breast cancer. Adjuvant chemotherapy reduces the risk of breast cancer death in both node positive and node negative cancers [18]. Since the treatment carries a non-trivial risk of serious complications, evidence-based guidelines often strongly recommend that the patient's risk of cancer recurrence and death (based upon cancer grade and stage) play an important role in determining who should receive chemotherapy [19]. However, breast cancer frequently behaves like a chronic disease, with events occurring over a decade or more, and there is tremendous variation in the risk of both breast cancer and non-breast cancer death across the breast cancer population.

Table 1 shows how the benefit of chemotherapy will vary according to both the baseline risk from the cancer itself and the degree of competing risk for mortality, even assuming a constant relative effect of treatment for all patients (relative risk reduction = 15%) and a constant absolute rate of serious treatment-related harm (15 events over 10 years per 1000 people treated). Based on these assumptions, and estimated survival rates for stages 1, 2 and 3 breast cancer [20], consistent with our previous work [6,9,10], those who are at very high risk of dying from the disease usually benefit substantially despite the risks of treatment-related harm, and not surprisingly, even when there is substantial competing risk. This is because when the baseline breast cancer risk is high the disease-specific risk overwhelms competing risks from comorbid illnesses, and an efficacious treatment produces a large amount of absolute benefit, far outweighing the small risk of treatment-related harm (see Table 1).

**Table 1:** Interactions between baseline risk, treatment-related harm (Rx-harm) and competing risk (CR) when chemotherapy reduces breast cancer mortality by 15%.

Risk of Breast CA Death	No Rx-harm or CR	Rx-harm (1.5% absolute rate) but no CR	Rx-harm & CR is:		
			
			Low (10%)	Moderate (25%)	High (50%)
(10-yr CA Mortality)	Absolute Risk Reduction/Number Needed to Treat				

Low (10%)	.015/67	0/8	-.002/-667§	-.004/-267§	-.007/-133§
Moderate (25%)	.038/27	.023/44	.019/53	.013/76	.004/267
High (50%)	.075/13	.060/17	.053/19	.041/24	.022/44

§ The negative sign denotes instances in which chemotherapy does net harm, meaning that the statistics represent absolute risk *increase *and number need to *harm*.

For patients with a more favorable prognosis, however, the absolute amount of benefit of the same treatment is much more modest, such that treatment-related harm and competing risk can greatly attenuate or reverse the net benefits of treatment. For example, a patient with a non-trivial 10% percent breast cancer mortality risk would appear to be a good candidate in the absence of other risks (number needed to treat [NNT] = 67); however, a small treatment-related risk would nullify their potential benefit and the presence of relatively modest competing risks would cause the treatment to result in net harm. Even for patients with a substantial 25% risk of breast cancer death in the absence of competing risks, a high rate of competing risk results in a greatly attenuated treatment-effect; the NNT increases from 44 (with no competing risks) to 267 (with a 50% 10-year competing risk of mortality). If patients with high competing risk also had twice the normal risk of treatment-related harm (and the risk of treatment-related harm is often influence by comorbid illness) then chemotherapy would result in net harm (number needed to harm [NNH] = 91).

Note that a 10-year competing risk of mortality of 50% is not extreme for breast cancer. Approximately a third of breast cancer patients are over the age of 70. The 10-year risk of competing mortality would be approximately 50% for a 70 year old at only slightly higher than average risk [21]. Indeed, a median age breast cancer patient (age 61) with asymptomatic class I CHF would also have approximately a 50% 10-year risk of competing mortality [22].

Table 1 demonstrates that the overall measured effectiveness of adjuvant chemotherapy in a clinical trial depends on the distribution of the competing and outcome risks of trial enrollees. By excluding older patient or those with comorbidities, [23,24] oncology trials enhance their likelihood of detecting treatment benefit, but their results are directly applicable only to patients with low competing risks. While enrolling older patients, and patients with multiple comorbidities would attenuate the treatment-effect in the summary results, it would still not provide the clinically useful knowledge about who to treat unless analyses included risk-based stratification, incorporating both competing and outcome risk.

### When Competing Risk is Correlated with Outcome-Risk

While the presence or absence of comorbidities should not substantially alter the likelihood of a more aggressive versus a more indolent form of cancer, in many circumstances, competing risk can be highly correlated with the disease-specific outcome-risk. For example, an implantable cardiac defibrillator (ICD) would be of most benefit in patients with a high risk for sudden cardiac death (SCD) but little risk for death from other causes [25], since implanting these devices (costly and not risk-free) in patients destined to die from non-arrhythmic causes is highly undesirable. However, the criteria used to identify eligible patients at high risk for sudden cardiac death (SCD), namely a left ventricular ejection fraction of 35% or less, also identifies patients at risk of cardiac death from pump failure [26,27]. Separating these risks has proven difficult, as factors that predict mortality from SCD also usually predict non-SCD mortality [28].

The Seattle Heart Failure Model (SHFM), developed on a database of pooled clinical trials consisting of 10,538 ambulatory patients with heart failure [29], predicts total mortality in patients with congestive heart failure based on easily obtainable clinical variables. Both SCD and pump failure death substantially increase across risk strata [30]. However, the ratio between SCD and pump failure death dramatically *decrease *in higher risk compared to lower risk patients; low risk patients with risk scores of zero have roughly a 7 to 1 SCD to pump failure death ratio, while this ratio was 1 to 2 in patients with risk scores of 4 [30].

**Figure 1:**
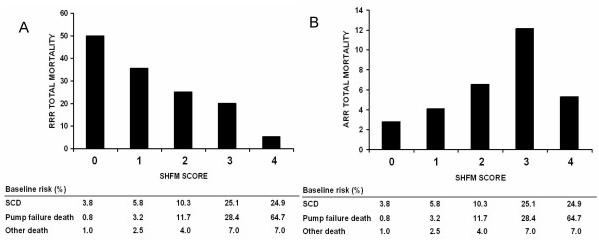
**Relative and Absolute Benefits for Implantable Cardiac Defibrillators (ICDs) Stratified by Total Mortality Risk**: These graphs show the relative (A) and absolute (B) benefit for ICDs assuming that the devices are 75% effective in preventing sudden cardiac death (SCD) but not at all effective in preventing pump failure. The risk ratio of SCD to pump failure death was empirically observed [30]. Note that both relative risk reduction decreases monotonically. Absolute risk reduction demonstrates a U-shaped benefit; benefit is low in low risk groups whose risk of SCD is low and in high risk groups who are suffer pump failure.

As Figure 1 demonstrates, these different ratios across risk strata can have important effects on the measured effectiveness of ICDs. As mortality risk increases, the relative risk reduction of ICDs dramatically decreases. This is because the relative risk reduction is inversely related to the ratio of preventable disease-specific (SCD) to non-preventable competing risk of mortality. More surprisingly, the absolute risk reduction across risk strata is described by a non-linear, inverted U-shaped function (Figure 1b). Intermediate risk patients are most likely to benefit, as low risk patients are unlikely to have an arrhythmic death even in the absence of treatment while benefit in the highest risk group is limited by the high rate of non-arrhythmic death. Folding in treatment-related harm could further amplify this treatment-effect heterogeneity, particularly if sicker patients were more prone to ICD-induced adverse events.

Indeed, the phenomena depicted in Figure 1 is consistent with a risk-stratified analysis of the Multicenter Automatic Defibrillator Implantation Trial (MADIT)-II [31,32]. While overall MADIT-II found a 31% reduction in all-cause two-year mortality associated with the ICD [31], the ICD did not appear to reduce all-cause mortality in either the very low risk group (defined as no more than 1 of multiple risk factors) or a very high risk group of patients (defined as those with blood urea nitrogen ≥ 50 mg/dL or serum creatinine ≥ 2.5 mg/dL) [32]. While the intermediate mortality risk group may represent the "sweet spot" for ICD therapy, more efficient targeting of these devices could presumably be achieved if risk factors for SCD that do not predict pump failure could be identified. Again, summary trial results would be quite dependent on the risk profile of the enrolled patients, and may be misleading in some circumstances.

### Summary

As seen in the clinical conditions above, variation in competing risk can cause variation in treatment-effect. Even for treatments with a constant efficacy, the apparent relative risk reduction of an intervention is directly related to the ratio between disease-specific and competing risk. When these two risk dimensions are correlated, increasing outcome-risk may not uniformly lead to increasing benefit – particularly when the primary outcome is a combination of disease-specific and competing events. Even when outcome and competing risks are not correlated, understanding how to treat individual patients can depend on the interplay between competing and outcome risks, and these effects can be greatly amplified by even small amounts of treatment-related harm, especially when those with higher competing risk are also at greater risk of harm from treatment. Calls for large simple clinical trials [33] with broad inclusion criteria, including older or complex patients, designed to provide results generalizable to "real-world" patients [3], have generally ignored the fact that reporting a summary treatment-effect based on the arithmetic mean across all patients may at times be misleading. The application of the results of meta-analysis without a careful consideration of clinical heterogeneity is also problematic [34]. Since treatment benefit depends on the ratio between competing and outcome risks, it may be necessary to stratify these real world effectiveness trials along these two important risk dimensions, as in Table 1, or to account for these risks using appropriate multivariable models.

Some might point out that thoughtful and experienced clinicians attempt to do this in clinical practice, such that overall clinical trial results can still be customized at the bedside. Doctors specializing in prostate cancer, for example, are known to apply the so-called 10-year rule [35], an implicit assessment based on age and co-morbidity, whereby aggressive therapy might be offered to patients likely to live long enough to benefit. However, physicians in general (and oncologists specifically) are prognostically inaccurate and systematically over-optimistic, when estimating overall life expectancy [36,37]. In addition to being inaccurate, implicit clinical judgment is an inadequate basis for clinical practice in an era where decisions are expected to conform to guidelines and will be evaluated based on performance measures. While tools which help formally assess comorbidities and competing risks may be helpful [38], considering and comparing patient-specific competing risks to patient-specific disease-specific risks adds a dimension of complexity likely to render simple bedside heuristics inadequate since these may be determined by different or similar factors, and particularly where the time course for benefits and harms of therapies can vary. Further, when results of clinical trials themselves are aggregated across patients with greatly varying disease-specific and competing risks, the underlying treatment-effect that should be incorporated in to any decisional framework at the bedside may be totally obscure.

## Conclusion

In order to build an evidence-base that can support guidelines for patients who have multiple diseases simultaneously, relaxing clinical trial eligibility criteria to include older and complex patient must be accompanied by analyses that examine how a treatment's net benefit varies by an individual's disease-specific risk, chance of treatment-related harm, and competing risks. Research is needed to develop and test reliable ways to capture competing risk for different conditions [35,39], to develop sound methodologies to examine treatment-effects across multiple dimensions of risk, to develop a consensus to standardize analytic approaches and to identify which circumstances and clinical conditions these more complex analytic approaches might be justified and necessary.

## Competing interests

The authors declare that they have no competing interests.

## Authors' contributions

All authors contributed equally to the development of the concepts in this manuscript. DMK had the initial idea for the manuscript and prepared the first draft based on contributions from all authors. All authors participated in manuscript revision.
